# Complete genome sequence of endophytic nitrogen-fixing *Klebsiella variicola* strain DX120E

**DOI:** 10.1186/s40793-015-0004-2

**Published:** 2015-05-08

**Authors:** Li Lin, Chunyan Wei, Mingyue Chen, Hongcheng Wang, Yuanyuan Li, Yangrui Li, Litao Yang, Qianli An

**Affiliations:** 1 Key Laboratory of Sugarcane Biotechnology and Genetic Improvement (Guangxi), Ministry of Agriculture; Guangxi Key Laboratory of Sugarcane Genetic Improvement; Sugarcane Research Institute, Guangxi Academy of Academy of Agricultural Sciences; Sugarcane Research Center, Chinese Academy of Agricultural Sciences, Nanning, China; 2 State Key Laboratory for Conservation and Utilization of Subtropical Agro-bioresources, Guangxi University, Nanning, China; 3 State Key Laboratory of Rice Biology, Institute of Biotechnology, Zhejiang University, Hangzhou, China; 4 Microbiology Research Institute, Guangxi Academy of Academy of Agricultural Sciences, Nanning, China

**Keywords:** Endophyte, Klebsiella variicola, Klebsiella pneumoniae, Nitrogen fixation, Pathogenicity, Plant growth-promoting bacteria, Sugarcane

## Abstract

*Klebsiella variicola* strain DX120E (=CGMCC 1.14935) is an endophytic nitrogen-fixing bacterium isolated from sugarcane crops grown in Guangxi, China and promotes sugarcane growth. Here we summarize the features of the strain DX120E and describe its complete genome sequence. The genome contains one circular chromosome and two plasmids, and contains 5,718,434 nucleotides with 57.1% GC content, 5,172 protein-coding genes, 25 rRNA genes, 87 tRNA genes, 7 ncRNA genes, 25 pseudo genes, and 2 CRISPR repeats.

## Introduction

The species *Klebsiella variicola * was classified in 2004 and consisted of clinical and plant-associated isolates [1].The species *K. singaporensis * was classified in 2004 based on a single soil isolate [2] and was recently identified as a later junior heterotypic synonym of *K. variicola *[3]. *K. variicola * is able to fix N_2_[1]. *K. variicola * strain At-22, one of the dominant bacteria in the fungus gardens of leaf-cutter ants, provides nitrogen source by N_2_ fixation [4] and carbon source by degrading leaf polymers to the ant-fungus symbiotic system [5]. Former *K. pneumoniae * strain 342 (Kp342), which is phylogenomically close to strain At-22 [6],[7] and has been identified as a strain of *K. variicola *[3], is able to colonize in plants and to provide small but critical amounts of fixed nitrogen to plant hosts [8].

*K. variicola * strain DX120E was isolated from roots of sugarcane grown in Guangxi, the major sugarcane production area in China [9]. It is able to colonize in sugarcane roots and shoots, to fix N_2_ in association with sugarcane plants, and to promote sugarcane growth [10], and thus shows a potential as a biofertilizer. Here we present a summary of the features of the *K. variicola * strain DX120E (=CGMCC 1.14935) and its complete genome sequence, and thus provide a genetic background to understand its endophytic lifestyle, plant growth-promoting potentials, and similarities and differences to other plant-associated and clinical *K. variicola * isolates.

## Organism information

### Classification and general features

**Figure 1:**
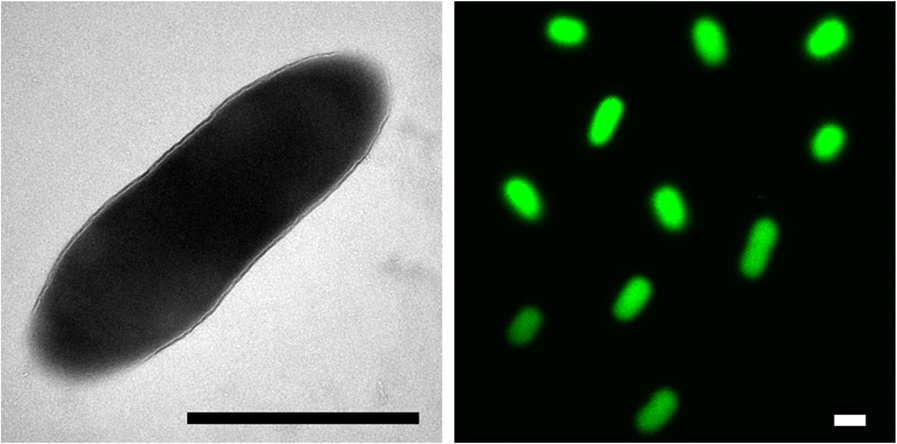
Morphology of *Klebsiella variicola* DX120E cells. Transmission electron micrograph (left) shows a DX120E cell stained by uranyl acetate; laser scanning confocal micrograph (right) shows DX120E cells tagged by green fluorescent protein. The scale bars represent 1 μm.

*K. variicola * strain DX120E is a Gram-negative, non-spore-forming, non-motile rod (Figure 1). It grows aerobically but reduces N_2_ to NH_3_ at a low pO_2_. It is able to grow and fix N_2_ on media containing 10% (w/v) cane sugar or sucrose. It forms circular, convex, smooth colonies with entire margins on the solid high-sugar content media. It grows best around 30°C and pH 7 (Table 1).

**Figure 2:**
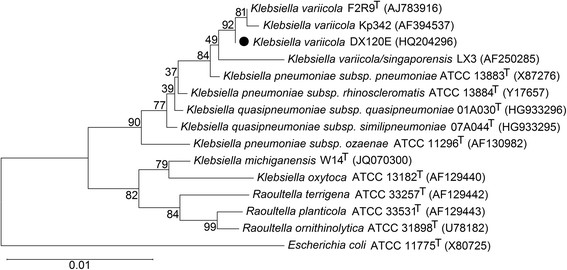
Phylogenetic tree of 16S rRNA gene sequences showing the position of *Klebsiella variicola* strain DX120E (●). *Escherichia coli* ATCC 11775^T^ is used as an outgroup. The sequences were aligned with the CLUSTAL W program and were constructed with the neighbor-joining algorithm and the Kimura 2-parameter model integrated in the MEGA 5.2 program [22]. The phylogenetic tree was tested with 1,000 bootstrap replicates. Bootstrap values are shown at the nodes. The GenBank accession numbers of the sequences are indicated in parentheses. The scale bar represents a 1% nucleotide sequence divergence. Note that the genome of strain DX120E, F2R9^T^ (DSM 15968^T^), or Kp342 contains eight copies of 16S rRNA genes; these gene sequences are generally not identical but phylogenetically grouped together (data not shown). The sequence of DX120E (HQ204296) used to construct the phylogenetic tree is identical to the sequence of locus tag KR75_09260 (CP009274:1935034–1936587).

Phylogenetic analysis of the 16S rRNA gene sequences from strain DX120E and strain Kp342, the type strains of the species in the genera *Klebsiella * and *Raoultella *, and the type strain of the type species of the type genus of the family *Enterobacteriaceae * (*Escherichia coli * ATCC11775^T^) showed that *K. variicola * strains (type strain F2R9, Kp342, DX120E and LX3) were most closely related and formed a monophyletic group with *K. pneumoniae * and *K. quasipneumoniae * (Figure 2).

Like typical members in the genera *Klebsiella *, *K. variicola * DX120E utilizes alanine, arabinose, D-arabitol, L-aspartate, D-cellobiose, citrate, D-fructose, L-fucose, D-galactose, gentiobiose, glucose, glycerol, myo-inositol, lactate, lactose, malate, maltose, D-mannitol, D-mannose, D-melibiose, L-proline, D-raffinose, L-rhamnose, L-serine, D-sorbitol, sucrose, and D-trehalose [23]. DX120E does not utilize adonitol (also known as ribitol), which is a distinctive characteristic from *K. pneumoniae *[1].

## Genome sequencing information

### Genome project history

*K. variicola * DX120E was selected for sequencing because it is a plant growth-promoting endophyte [10]. Its 16S rRNA gene sequence is deposited in GenBank under the accession number HQ204296. Its genome sequences are deposited in GenBank under the accession numbers CP009274, CP009275, and CP009276. A summary of the genome sequencing project information and its association with MIGS version 2.0 [11] is shown in Table 2.

**Table 1:** **Classification and general features of** ***Klebsiella variicola*** **strain DX120E according to the MIGS recommendations**[11]

**MIGS ID**	**Property**	**Term**	**Evidence code**^**a**^
	Classification	Domain *Bacteria*	TAS [12]
		Phylum *Proteobacteria*	TAS [13]
		Class *Gammaproteobacteria*	TAS [14],[15]
		Order *Enterobacteriales*	TAS [16]
		Family *Enterobacteriaceae*	TAS [17],[18]
		Genus *Klebsiella*	TAS [18],[19]
		Species *Klebsiella variicola*	TAS [1],[20]
		Type strain:F2R9^T^ (ATCC BAA-830 = DSM 15968)	TAS [1]
	Gram stain	Negative	IDA
	Cell shape	Rod	IDA
	Motility	Non-motile	IDA
	Sporulation	Non-sporulating	IDA
	Temperature range	4–40°C	IDA
	Optimum temperature	28–32°C	IDA
	pH range; Optimum	3.5–8.5; 7.0	IDA
	Carbon source	Sucrose, citrate, fructose, galactose, glucose, lactose, malate, maltose, mannitol, mannose, rhamnose, & sorbitol	IDA
MIGS-6	Habitat	Soil, plants	IDA
MIGS-6.3	Salinity	0 – 5% NaCl (w/v)	IDA
MIGS-22	Oxygen requirement	Aerobic	IDA
MIGS-15	Biotic relationship	Free-living, endophytic	IDA
MIGS-14	Pathogenicity	Not reported	
MIGS-4	Geographic location	Daxin, Guangxi, China	TAS [9]
MIGS-5	Sample collection	2008	TAS [9]
MIGS-4.1 MIGS-4.2	Longitude	107°20’E	NAS
	Latitude	22°80’N	NAS
MIGS-4.3	Depth	0.1 – 0.2 m below the surface	IDA
MIGS-4.4	Altitude	320 m	NAS

^a^Evidence codes – IDA: Inferred from Direct Assay; TAS: Traceable Author Statement (i.e., a direct report exists in the literature); NAS: Non-traceable Author Statement (i.e., not directly observed for the living, isolated sample, but based on a generally accepted property for the species, or anecdotal evidence). These evidence codes are from the Gene Ontology project [21].

**Table 2:** **Genome sequencing project information for** ***Klebsiella variicola*** **strain DX120E**

**MIGS ID**	**Property**	**Term**
MIGS-31	Finishing quality	Finished
MIGS-28	Libraries used	PacBio 4 –10Kb library
		Illumina 500 bp library
MIGS-29	Sequencing platforms	PacBio RS II
		Illumina HiSeq 2000
MIGS-31.2	Fold coverage	PacBio 96 ×
		Illumina 106 ×
MIGS-30	Assemblers	HGAP in smrtanalysis-2.1.1SOAPdenovo 2.04
MIGS-32	Gene calling method	GeneMarkS+
	Locus Tag	KR75
	Genbank ID	CP009274 (Chromosome)
		CP009275 (plasmid pKV1)
		CP009276 (plasmid pKV2)
	Genbank Date of Release	January 1, 2015
	GOLD ID	Gi0078577
	BIOPROJECT	PRJNA259590
MIGS 13	Source Material Identifier	CGMCC 1.14935
	Project relevance	Agriculture, plant-microbe interactions

### Growth conditions and DNA isolation

*K. variicola * DX120E was grown in liquid Luria-Bertani (LB) medium at 30°C to early stationary phase. The genome DNA was extracted from the cells by using a TIANamp bacterial DNA kit (Tiangen Biotech, Beijing, China). DNA quality and quantity were determined with a Nanodrop spectrometer (Thermo Scientific, Wilmington, USA).

### Genome sequencing and assembly

The genome DNA of *K. variicola * DX120E was constructed into a 4 – 10 kb insert library and sequenced by the Pacific Biosciences’ (PacBio) Single Molecule, Real-Time (SMRT) sequencing technology [24] at the Duke University Genome Sequencing & Analysis Core Resource. Sequencing was run on single SMRT cell and resulted in 91,190 high-quality filtered reads with an average length of 6,196 bp. High-quality read bases were assembled by the Hierarchical Genome Assembly Process (HGAP) with smrtanalysis-2.1.1. The resulting draft genome consisted of 5,719,400 nucleotides and 5 contigs.

The genome DNA of *K. variicola * DX120E was also constructed into a 500-bp insert library and sequenced by an Illumina HiSeq 2000 sequencing system at BGI Tech, Shenzhen, China. The Illumina HiSeq 2000 sequencing resulted in 6,699,933 high-quality filtered reads with an average length of 90 bp. The sequencing data were assembled by the Short Oligonucleotide Analysis Package (SOAPdenovo 2.04) [25]. The resulting draft genome consisted of 5,695,362 nucleotides and 27 scaffolds.

The two draft genomes were aligned by Mauve [26]. The Illumina scaffold 1 bridged the PacBio contig 1 and contig 2; the Illumina scaffold 3 bridged the PacBio contig 1, contig 2, and contig 3; the Illumina scaffold 11 bridged the circular PacBio contig 4; the Illumina scaffold 16 bridged the circular PacBio contig 5. The genome sequencing was completed by PCR and Sanger sequencing to close the contig gaps of the PacBio-sequenced genome.

### Genome annotation

Automated genome annotation was completed by the NCBI Prokaryotic Genome Annotation Pipeline. Product description annotations were obtained by searching against the KEGG, InterPro, and COG databases. Genes with signal peptides were predicted by SignalP [27]. Genes with transmembrane helices were predicted by TMHMM [28]. Genes for tRNA were found by tRNAScanSE [29]. Ribosomal RNAs were found by BLASTN vs. ribosomal RNA databases; 5S rRNA hits were further refined by Cmsearch [30]. Thirteen disrupted genes were replaced by the complete gene sequences obtained from the Illumina HiSeq 2000 sequencing.

### Genome properties

**Figure 3:**
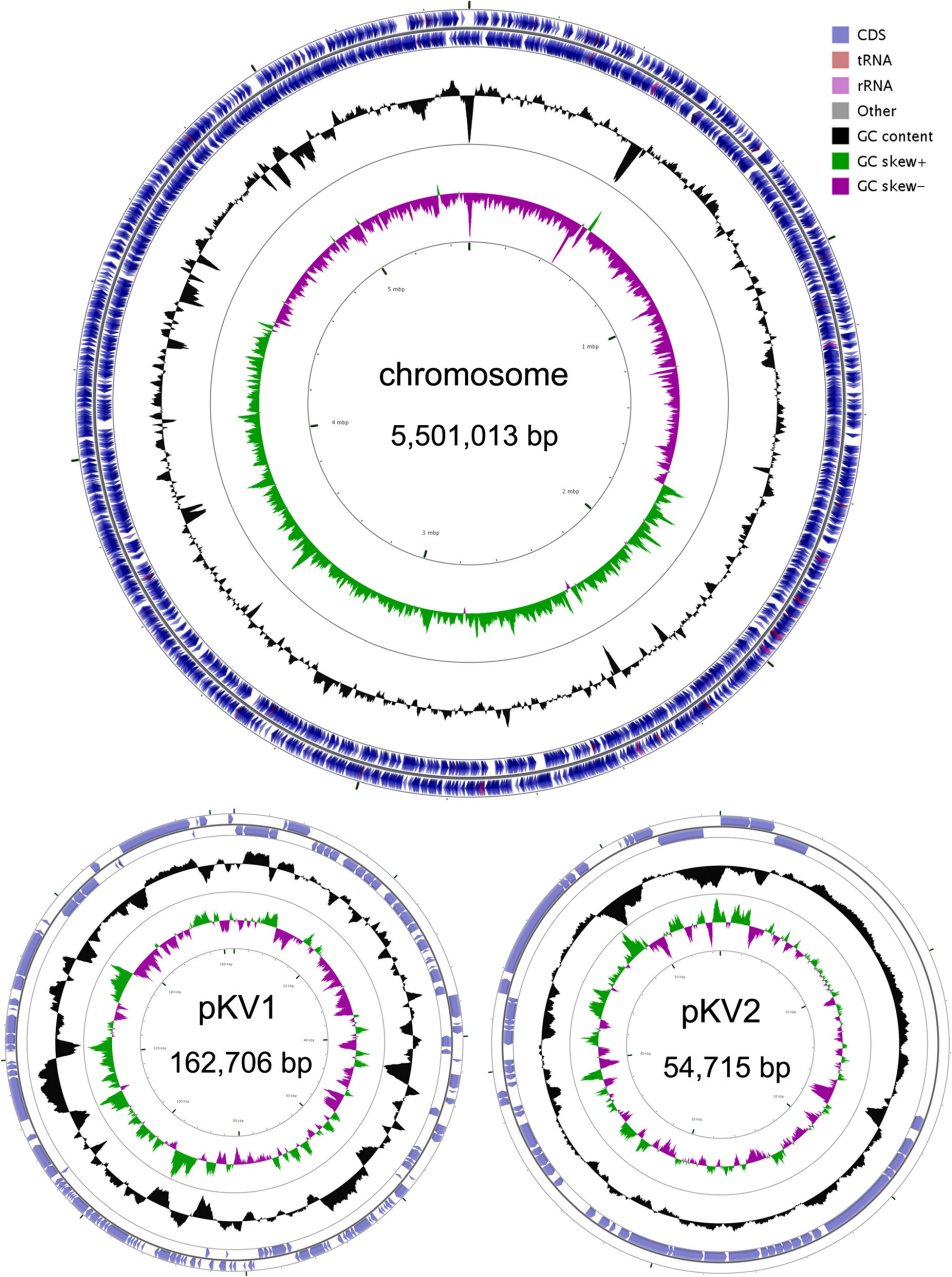
Circular map of the chromosome and plasmids of *Klebsiella variicola* strain DX120E. From outside to the center: genes on forward strand, genes on reverse strand, GC content, GC skew. Circular map was generated by CGView [31].

The genome of *K. variicola * DX120E contains one circular chromosome and two plasmids (pKV1 and pKV2) (Table 3, Figure 3). The chromosome contains 5,501,013 nucleotides with 57.3% G + C content. The plasmid pKV1 contains 162,706 nucleotides with 50.7% G + C content. The plasmid pKV2 contains 54,715 nucleotides with 53.1% G + C content. The genome contains 5,316 predicted genes, 5,172 protein-coding genes, 119 RNA genes (25 rRNA genes, 87 tRNA genes, and 7 ncRNA genes), 25 pseudo genes, and 2 CRISPR repeats. The chromosome, pKV1, and pKV2 contain 4990, 131, and 51 protein-coding genes with coding density of 87.3%, 74.2%, and 83.9%, respectively. Among the 5,172 protein-coding genes, 4,511 genes (87.2%) have been assigned functions, while 661 genes (12.8%) have been annotated as hypothetical or unknown proteins (Table 4). The distribution of genes into COGs functional categories is presented in Table 5.

**Table 3:** Summary of genome: one chromosome and two plasmids

**Label**	**Size (bp)**	**Topology**	**INSDC identifier**	**RefSeq ID**
Chromosome	5,501,013	Circular	CP009274.1	NZ_CP009274.1
Plasmid pKV1	162,706	Circular	CP009275.1	NZ_CP009275.1
Plasmid pKV2	54,715	Circular	CP009276.1	NZ_CP009276.1

**Table 4:** Genome statistics

**Attribute**	**Value**	**% of total**
Genome size (bp)	5,718,434	100
DNA coding (bp)	4,930,539	86.22
DNA G + C (bp)	3,265,303	57.10
DNA scaffolds	3	100
Total genes	5,316	100
Protein-coding genes	5,172	97.29
RNA genes	112	2.12
Pseudo genes	25	0.47
Genes with function prediction	4,623	87.00
Genes assigned to COGs	4,398	82.73
Genes with Pfam domains	4,631	87.11
Genes with signal peptides	526	9.89
Genes with transmembrane helices	1,289	24.25
CRISPR repeats	2	0.04

**Table 5:** Number of genes associated with general COG functional categories

**Code**	**Value**	**% age**	**Description**
J	198	3.83	Translation, ribosomal structure and biogenesis
A	1	0.02	RNA processing and modification
K	489	9.45	Transcription
L	159	3.07	Replication, recombination and repair
B	1	0.02	Chromatin structure and dynamics
D	43	0.83	Cell cycle control, cell division, chromosomepartitioning
V	71	1.37	Defense mechanisms
T	235	4.54	Signal transduction mechanisms
M	260	5.03	Cell wall/membrane biogenesis
N	62	1.20	Cell motility
U	111	2.15	Intracellular trafficking and secretion
O	158	3.05	Posttranslational modification, protein turnover, chaperones
C	342	6.61	Energy production and conversion
G	583	11.27	Carbohydrate transport and metabolism
E	538	10.40	Amino acid transport and metabolism
F	102	1.97	Nucleotide transport and metabolism
H	215	4.16	Coenzyme transport and metabolism
I	130	2.51	Lipid transport and metabolism
P	344	6.65	Inorganic ion transport and metabolism
Q	112	2.17	Secondary metabolites biosynthesis, transport and catabolism
R	541	10.46	General function prediction only
S	414	8.00	Function unknown
-	774	14.97	Not in COGs

The total is based on the total number of protein coding genes in the genome.

### Insights from the genome sequence

The genome of *K. variicola * DX120E contains genes contributing to multiple plant-beneficial functions. In accordance with previously detected N_2_ fixation, indole-3-acetic acid production, siderophore production, and phosphate solubilization [9], the genome of *K. variicola * DX120E contains *nif* cluster, indole-3-pyruvate decarboxylase, siderophore enterobactin synthesis genes (*entABCDEF*) and enterobactin exporter gene (*entS*), and pyrroloquinoline quinone synthesis genes (*pqqBCDEF*) contributing to these functions. Moreover, the genome of *K. variicola * DX120E contains the *budABC* operon for the synthesis of acetoin and 2,3-butanediol [32], and thus may induce plant systemic resistance to pathogens [33].

DX120E contains plasmids similar to those in *Klebsiella * relatives. The plasmid pKV1 is most similar to the plasmid pKp5-1 of the *K. pneumoniae * strain 5–1 (Kp5-1) [34] with a 97% identity of 56% coverage (Additional file 1: Figure S1); the similar regions mainly encode transposase/recombinases and proteins functioning in plasmid replication, partitioning, and conjugal transfer. The plasmid pKV2 is most similar to the plasmid pKOXM1C of the *K. oxytoca * strain M1 with a 96% identity of 89% coverage (Additional file 2: Figure S2); the similar regions mainly encode proteins for plasmid partitioning and phage functions.

The genome of *K. variicola * DX120E has high average nucleotide identities (ANI) [35] about 99% to the available genomes of *K. variicola * strains DSM 15968^T^, At-22, Bz19, and Kp342. Bz19 was isolated from faeces of a hospitalized patient [6]. The plant-beneficial strain Kp342 is able to infect mouse organs, although it is less virulent than typical clinical *K. pneumoniae * isolates [36]. Kp5-1, which has the plasmid pKp5-1 close to pKV1, is a cotton pathogen causing boll-rot disease [34]. The genome of strain Kp5-1 has ANI values about 99% to the genomes of the known *K. variicola * strains and thus belongs to *K. variicola *. These drive concerns about potential pathogenicity of DX120E to animals and plants. Therefore, DX120E’s pathogenic potentials to animals and plants should be determined before using DX120E as a biofertilizer in the field.

## Conclusions

The complete genome sequence of *K. variicola * DX120E provides the genetic background for understanding the bacterial mechanisms to adapt endophytic life and to promote plant growth. The high degree of whole-genome and plasmid similarities between DX120E and phytopathogenic and clinical *Klebsiella * isolates suggests the risk of using DX120E as a biofertilizer. The available genome sequences of the *K. variicola * strains allow an in-depth comparative analysis to understand the subtle pathogenicity mechanisms of the pathogens and to predict pathogenic risks for the plant-beneficial strain DX120E.

## Competing interests

The authors declare that they have no competing interests.

## Authors' contribution

LL did the microbiological studies and obtained the organism information; CW assembled the Illumina sequencing data; MC assembled the PacBio sequencing data; HW and YYL completed the genome analysis; YRL, LY, and QA designed the study and wrote the manuscript. All authors read and approved the final manuscript.

## Additional files

## Supplementary Material

Additional file 1: Figure S1. Comparison of plasmid pKV1 of *Klebsiella variicola* strain DX120E with plasmid pKp5-1 of *K. pneumoniae* strain 5–1. Click here for file

Additional file 2: Figure S2. Comparison of plasmid pKV2 of *Klebsiella variicola* strain DX120E with plasmid pKOXM1C of *K. oxytoca* strain M1. Click here for file
