# Supplementary material for: Complete genome sequence of endophytic nitrogen-fixing Klebsiella variicola strain DX120E
Source: Stand Genomic Sci. 2015 May 8;10:22. doi: 10.1186/s40793-015-0004-2 (PMC4511632; doi:10.1186/s40793-015-0004-2)
Supplement: Additional file 1: Figure S1. — Comparison of plasmid pKV1 of Klebsiella variicola strain DX120E with plasmid pKp5-1 of K. pneumoniae strain 5–1. [file s40793-015-0004-2-S1.pdf]

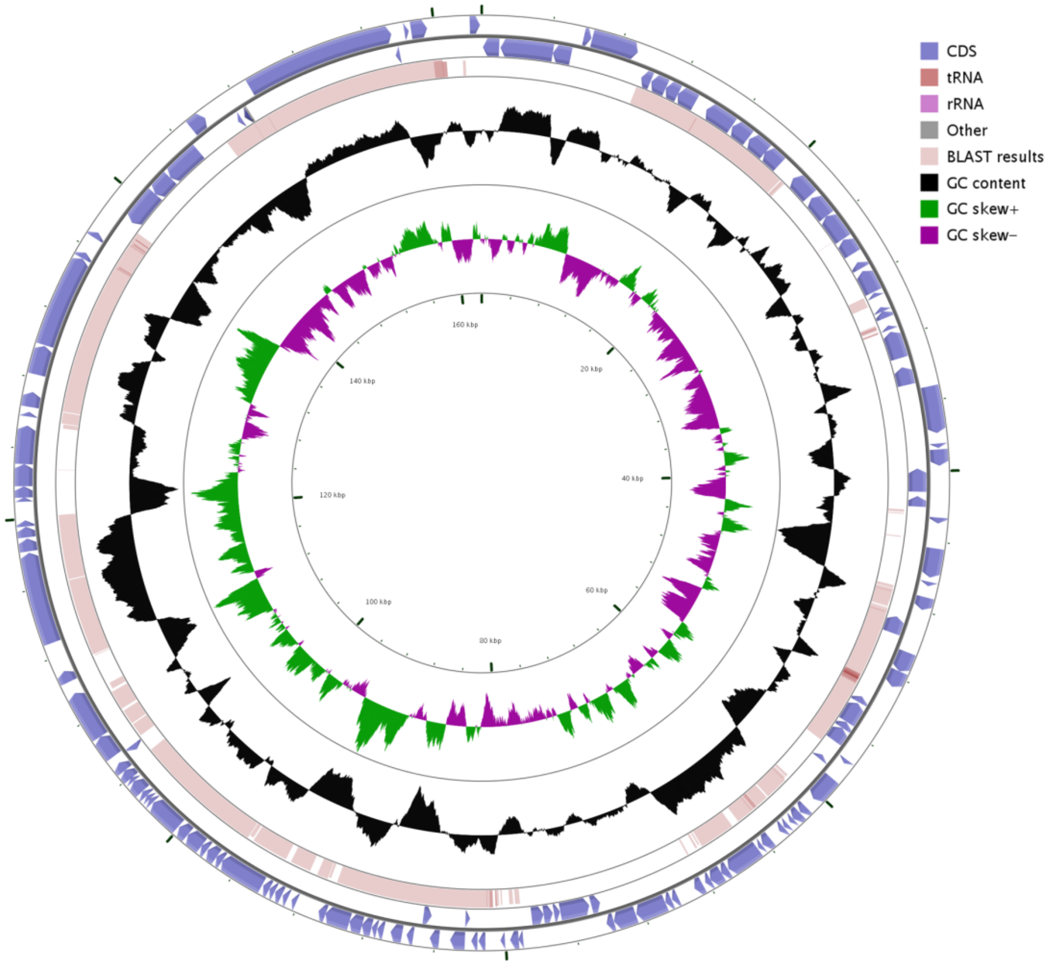

Figure S1. Comparison of plasmid pKV1 of *Klebsiella variicola* strain DX120E with plasmid pKp5-1 of *K. pneumoniae* subsp. *pneumoniae* strain 5-1. From outside to the center: genes on forward strand, genes on reverse strand, blast hits obtained from blastn search of plasmid pKp5-1, GC content, GC skew.

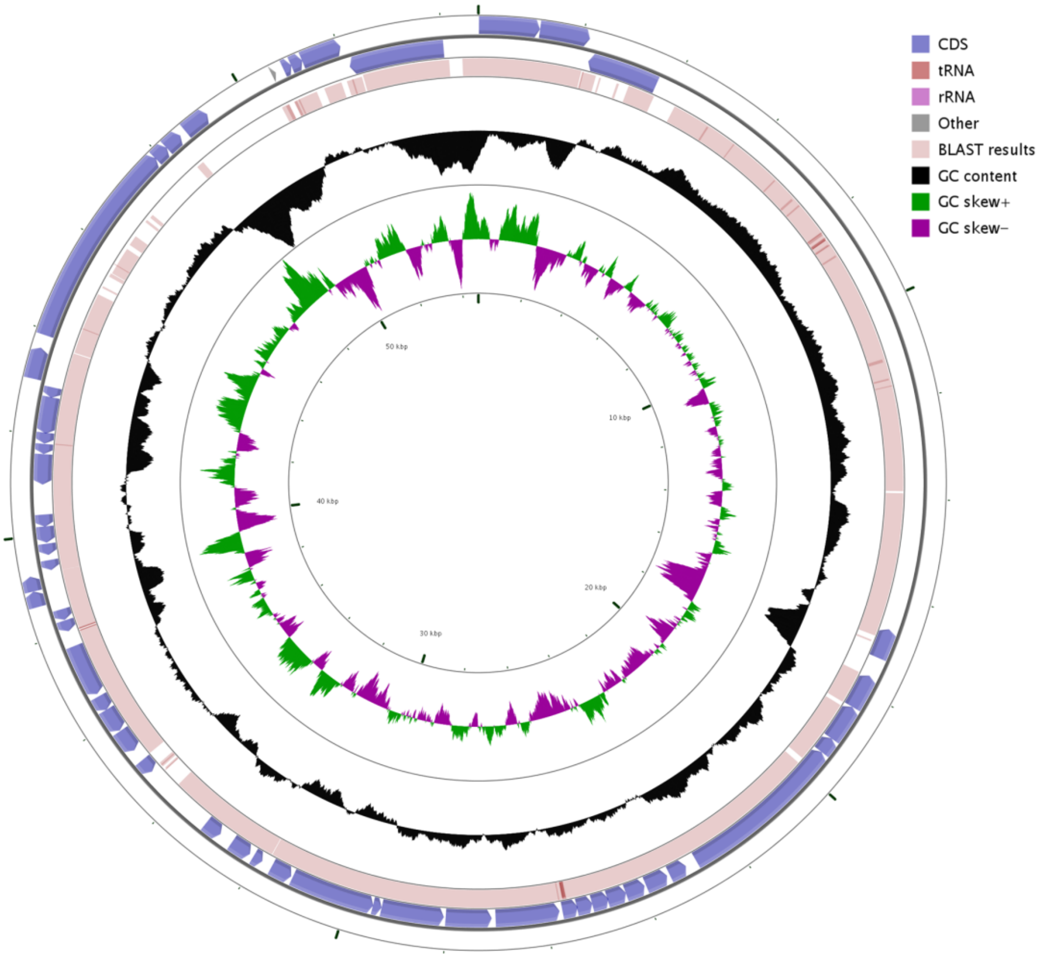

Figure S2. Comparison of plasmid pKV2 of *Klebsiella variicola* strain DX120E with plasmid pKOXM1C of *K. oxytoca* strain M1. From outside to the center: genes on forward strand, genes on reverse strand, blast hits obtained from blastn search of plasmid pKOXM1C, GC content, GC skew.
